# Probiotic Properties of *Bacillus proteolyticus* Isolated From Tibetan Yaks, China

**DOI:** 10.3389/fmicb.2021.649207

**Published:** 2021-08-17

**Authors:** Zhibo Zeng, Xiaoling He, Feiran Li, Yan Zhang, Zonghao Huang, Yaping Wang, Kun Li, Yuhua Bao, Mudassar Iqbal, Muhammad Fakhar-e-Alam Kulyar, Jiakui Li

**Affiliations:** ^1^College of Veterinary Medicine, Huazhong Agricultural University, Wuhan, China; ^2^College of Veterinary Medicine, Nanjing Agricultural University, Nanjing, China; ^3^Tibet Autonomous Region Biological Drug Manufacturing Plant, Lhasa, China; ^4^Faculty of Veterinary and Animal Sciences, The Islamia University of Bahawalpur, Bahawalpur, Pakistan; ^5^College of Animals Husbandry and Veterinary Medicine, Tibet Agricultural and Animal Husbandry University, Linzhi, China

**Keywords:** yaks, antioxidant capacity, *Bacillus proteolyticus*, *Bacillus amyloliquefaciens*, probiotics

## Abstract

Yaks (*Bos grunniens*) live primarily in high-altitude hypoxic conditions and have a unique intestinal micro-ecosystem, remarkable adaptability, and strong climatic resistance. Accumulating evidence revealed the importance of probiotics in host metabolism, gut microbiota, growth performance, and health. The goal of this study was to screen out probiotics with excellent probiotic potential for clinical application. In this study, four strains of *Bacillus*, i.e., *Bacillus proteolyticus* (named Z1 and Z2), *Bacillus amyloliquefaciens* (named J), and *Bacillus subtilis* (named K), were isolated and identified. Afterward, their probiotic potential was evaluated. Antioxidant activity tests revealed that Z1 had the highest DPPH and hydroxyl radical scavenging activity, whereas Z2 had higher reducing power and inhibited lipid peroxidation. Additionally, the antibacterial testing revealed that all strains were antagonistic to three indicator pathogens, *Escherichia coli* C83902, *Staphylococcus aureus* BNCC186335, and *Salmonella enteritidis* NTNC13349. These isolates also had a higher hydrophobicity, autoaggregation, and acid and bile tolerance, all of which helped to survive and keep dangerous bacteria out of the host intestine. Importantly, all strains could be considered safe in terms of antibiotic susceptibility and lack of hemolysis. In conclusion, this is the first study to show that *B. proteolyticus* and *B. amyloliquefaciens* isolated from yaks have probiotic potential, providing a better foundation for future clinical use.

## Introduction

Reactive oxygen species (ROS) is a single-electron reduction product of oxygen in the body, including superoxide anion, hydroxyl radical, and hydrogen peroxide (Mukherjee et al., 2014). Previous studies have shown that ultraviolet radiation, inflammatory cytokines, ionizing radiation, and chemicals are the primary sources of exogenous ROS production and intracellular oxidative metabolism that induce endogenous free radicals (Moné et al., 2011). Generally, there are antioxidant defense systems, including antioxidant enzymes and non-enzymatic antioxidants, in most cells to eliminate free radicals, which are constantly generated. Oxidative stress is a series of damage processes of lipid peroxidation, protein denaturation, and DNA hydroxylation, all of which are primarily produced by oxygen free radicals when the body is unable to remove them. Previous studies have shown that oxidative stress is closely related to cancer, parkinsonism, diabetes mellitus, and multiple cardiovascular diseases (Pires et al., 2019).

Additionally, oxidative stress can also reduce production performance and meat quality, resulting in severe economic losses to the breeding industry, although some synthetic antioxidants such as butylated hydroxytoluene and tert-butylated hydroxyanisole are widely used to relieve oxidative stress (Hossain et al., 2020). These antioxidants are not currently recommended due to hepatic injury and carcinogenicity. Therefore, increasing research is devoted to finding safer and more natural antioxidants to alleviate the adverse effects of oxidative damage.

Increasing evidence suggested that probiotics have many health benefits to the host, such as maintaining intestinal flora balance, modulating immune responses, improving growth performance, and antimicrobial activities. Additionally, several recent studies have suggested the ability of the probiotic in enhancing antioxidant properties. Wang et al. reported that *Bacillus amyloliquefaciens* SC06 could significantly increase the antioxidant capacity of porcine intestinal epithelial cells to alleviate the oxidative stress induced by hydrogen peroxide (Wang et al., 2019). Probiotic representatives of species *Bacillus subtilis* and *Lactobacillus casei* can scavenge free radicals (*in vitro*) and reduce oxidative damage by improving lipid metabolism and reducing lipid peroxidation (Wang et al., 2018).

The Tibetan Plateau (average elevation 4,000 m) is the world’s highest plateau. Low oxygen partial pressure and intense UV radiation characterize the frigid environment, which changes greatly from day to night (Li et al., 2020). Yak is an indigenous breed of the Qinghai–Tibet Plateau, and some researchers suggest that yaks have inhabited the region for millions of years (Li et al., 2015). Despite the fact that the hard-living environment might cause oxidative damage to the animal’s body, the yak has thoroughly adapted to the harsh conditions of the Tibetan plateau. Therefore, microorganisms living in the intestines of yaks may also have antioxidant properties (Li et al., 2019a). However, there have been few studies on the oxidation resistance of probiotics in yaks. As a result, the goal of the present study was to evaluate if *Bacillus* isolated from yak possesses antioxidant capabilities.

## Materials and Methods

### Isolation and Identification of *Bacillus* Strains

The fecal samples used in the present study were collected from the yaks of the Tibet Autonomous Region. The feces (2 g) were mixed with sterile phosphate-buffered saline and boiled for 15 min at 80°C. The supernatant (0.1 ml) was plated out in triplicate over Luria–Bertani (LB) agar and cultured for 24 h at 37°C under aerobic conditions. The suspected *Bacillus* strains with milky white were selected for purification and cultivation until the colony morphology was nearly comparable. Suspected strains were also tested by Gram staining and bacterial biochemical kits (Qingdao Haibo Biotechnology Co.). We used the technique of Wang et al. (2018) for conducting 16S rRNA sequencing to identify the bacteria species further. The isolated strains’ genomic DNA was extracted using Bacterial DNA Isolation Kit (Tiangen Biotech Co., Ltd.) and 16S rRNA via universal PCR primers. Finally, the PCR products were transferred to Qingke Biotech Company (Wuhan, China) for sequencing. MEGA 6 software was used to perform BLAST analysis and build a phylogenic tree using the acquired gene sequences. Furthermore, new evidence suggested using draft genome sequencing or another suitable approach.

### PCR Amplification of Antimicrobial Resistance Genes

To detect whether the four isolates carry the resistance genes, all strains had tested for *tet(K), tet(L), tet(M), tet(O), vanA,* and *vanB* genes (Table 1).

**TABLE 1:** PCR primers used in this study.

**Target gene**	**Primer pair**	**5′-3′,sequence**	**References**
*tet(K)*	*tet(K)-F tet(K)-R*	TTAGGTGAAGGGTTAGGTCC GCAAACTCATTCCAGAAGCA	Aarestrup et al., 2000
*tet(L)*	*tet(L)-F tet(L)-R*	CATTTGGTCTTATTGGATCG ATTACACTTCCGATTTCGG	Aarestrup et al., 2000
*tet(M)*	*tet(M)-F tet(M)-R*	GTTAAATAGTGTTCTTGGAG CTAAGATATGGCTCTAACAA	Aarestrup et al., 2000
*tet(O)*	*tet(O)-F tet(O)-R*	GATGGCATACAGGCACAGAC CAATATCACCAGAGCAGGCT	Aarestrup et al., 2000
*vanA*	*vanA-F vanA-R*	GGGAAAACGACAATTGC GTACAATGCGGCCGTTA	Dutka-Malen et al., 1995
*vanB*	*vanB-F vanB-R*	GTGCTGCGAGATACCACAGA CGAACACCATGCAACATTTC	Ramos-Trujillo et al., 2003

### Resistance to Hydrogen Peroxide

The hydrogen peroxide tolerance test is an important part of the antioxidant test (Asad et al., 1998). We designed and experimented, as previously reported by Eiamphungporn, to assess the isolated strains’ resistance to hydrogen peroxide, with few modifications (Eiamphungporn et al., 2003). The overnight cultures of isolated strains were inoculated at the level of 10^9^ CFU/ml in LB broth containing 0, 0.4, 0.6, 0.8, and 1.2 mM H_2_O_2_ at 37°C for 8 h. Afterward, the cultured bacteria suspension was diluted by using isotonic saline and 0.1 ml of bacterial diluent in triplicate on LB agar. After incubation at 37°C for 24 h in a constant temperature incubator, the bacteria populations were visually counted on LB agar to assess bacterial viability at various hydrogen peroxide concentrations.

### Hydroxyl Radical Scavenging Activity

The bacterial saline suspension (bacterial suspension) was incubated for 24 h and then centrifuged at 4°C for 10 min at 4,000 × *g*. The supernatant was discarded, and the remaining precipitate was then washed with sterile saline. After one more repeat, the bacterial suspension was allowed to be monitored at 600 nm on a UV spectrophotometer (UV1800, Shanghai AUCY Scientific Instrument Co., Ltd.) in order to obtain the value of 1.0.

The isolated strains were cultured for 24 h before being broken down for 20 min using an ultrasonic cell crusher (Shanghai Ji Pu Electronic Technology Co.) and centrifuged at 4°C for 10 min at 12,000 × *g* to obtain the bacteria-free extract. The sediment was discarded, and the supernatant was finally obtained.

The modified Fenton reaction method was used to evaluate the isolated strains’ hydroxyl radical scavenging activity (Areskogh and Henriksson, 2011). The reaction system of Fenton was 4.5 ml containing 1.0 ml of brilliant green (0.435 mM), 2.0 ml of ferrous sulfate (0.5 mM), 1.5 ml of hydrogen peroxide (3.0%, w/v), 1.0 ml of bacterial saline suspension and 1.0 ml of bacteria-free extract. The above reactants were mixed uniformly and then incubated at 37°C for 30 min in a thermostat water bath. The reaction mixture was centrifuged at 8,000 × *g* for 10 min at 4°C high-speed centrifuges (H2050R-1, Changsha, China). The absorbance of the resulting supernatant was monitored at 525 nm via a visible, ultraviolet spectrophotometer (UV1800, Shanghai AUCY Scientific Instrument Co., Ltd.). The hydroxyl radical scavenging activity (%) was calculated with the following formula:

$$\left[\frac{\left(A\,S-A\,0\right)}{A-A\,0}\right]\times 100\%$$

where A is the absorbance of the blank in the absence of H_2_O_2_, AS represents the absorbance of each sample, and A0 represents the absorbance of the supernatant without the samples.

### DPPH Radical Scavenging Activity

The method of Wu, with some modifications, was used to evaluate the DPPH radical scavenging activity of isolated strains (Wu et al., 2014). Precisely, the reaction mixture, including 2.0 ml of 0.4 mM DPPH solution (diluted with 95% ethanol), 1.0 ml of bacterial saline suspension and 1.0 ml of bacteria-free extract was placed in darkness for 30 min at room temperature. Afterwards, the absorbance of the isolated supernatant was measured at 517 nm after centrifugation at 8,000 × *g* for 10 min. The DPPH radical scavenging activity (%) was calculated as

$$\left[1-\frac{\left(A\,S-A\,B\right)}{A\,C}\right]\times 100\%$$

where AS represents the absorbance of the sample, AB represents the absorbance of the blank consisting of bacteria samples and ethanol and AC represents the absorbance of control consisting of DPPH solution and deionized water.

### Reducing Power

The reducing power of the isolated strains was determined according to Oyaizu with minor modifications (Oyaizu, 1988). Each isolated strain’s bacterial suspension (0.5 ml) and the bacteria-free extract (0.5 ml) were mixed with phosphate buffer solution (0.2 mol/L, pH 6.6) and 1% potassium ferricyanide (2.5 ml, m/v) and incubated for 20 min at 50°C. The reaction mixture was then quickly cooled in ice water before being terminated with 2.5 ml of 10% trichloroacetic acid (w/v). The mixture reaction was centrifuged at 3,000 × *g* for 10 min, then the obtained supernatant (2 ml) was mixed with 1 ml of ferric chloride (0.1% m/v) and 2 ml of deionized water. The absorbance of the mixture was measured at 700 nm after 10 min. Instead of the sample, deionized water was used to substitute the control group. The higher absorbance value in this assay indicated that the reducing power of the sample had improved.

$$\left(\frac{A\,S-A\,B}{A\,B}\right)\times 100\%$$

where AS represents the absorbance of the sample and AB represents the absorbance of the blank.

### Inhibition of Lipid Peroxidation

In the present study, the thiobarbituric acid (TBA) method was performed to assess the isolated strains to inhibit unsaturated linoleic acid peroxidation (Klimek et al., 1982). The linoleic acid emulsion (20 ml) was prepared by mixing linoleic acid (0.1 ml), Tween 20 (0.2 ml) and deionized water (19.7 ml). An aliquot of bacterial suspension (0.4 ml) and bacteria-free extract (0.4 ml) of isolated strains were mixed with 0.5 ml of phosphate buffer solution (0.02 mol/L, pH 7.4), 0.2 ml of 0.01% FeSO_4_ (m/v), 1 ml of linoleic acid emulsion, and 0.02 ml of 0.01% ascorbic acid (m/v) and incubated at 37°C for 12 h. After that, the reaction solution (2 ml) was mixed with 2 ml of 0.8% thiobarbituric acid (TBA, m/v), 0.2 ml of 0.4% Tert-butyl para-cresol (BHT, m/v), and 0.2 ml of 4% trichloroacetic acid (TCA, m/v). The above mixture was allowed to cool quickly on ice water after incubating at 100°C for 30 min. Simultaneously, the obtained supernatant was centrifuged again for 10 min with the same conditions, and then the absorbance of the supernatant was measured at 523 nm. The inhibition rate of linoleic acid peroxidation was calculated by using the following equation:

$$\left(1-\frac{A\,S}{A\,B}\right)\times 100\%$$

where AS represents the absorbance of the sample and AB represents the absorbance of the blank.

### Heat Tolerance, pH, and Bile Salt

The overnight cultures of isolated strains were centrifuged at 3,000 × *g* for 10 min and then washed three times with phosphate-buffered saline (PBS) for further experiments. The medium was adjusted to pH 7.2 by adding 1% trypsin (m/v) or pH 2.5 with a hydrochloric acid solution containing 1% pepsin (m/v). After incubation for 0, 1, and 2 h, the isolated strains’ viable cell number was counted using the plate count method. Also, the cultures were inoculated into LB broth containing different bile salts (0.1, 0.2, 0.3, 0.4, and 0.5%) and hydrochloric acid concentrations (pH 2.0, pH 3.0, pH 4.0, and pH 5.0). Also, the LB broth without bile salts and hydrochloric acid (pH 7.0) were used as a control. In order to test heat resistance, the bacterial suspensions were heated at 40, 50, 60, 70, 80, 90, and 100°C for 15 min. During this procedure, bacterial suspension without heat treatment was used as a control. At the end, the absorbance of the culture was measured at 600 nm to calculate the survival rate according to the following equation:

$$S\,u\,r\,v\,i\,v\,a\,l\,R\,a\,t\,e=\frac{\left(E\,x\,p\,e\,r\,i\,m\,e\,n\,t\,a\,l\,G\,r\,o\,u\,p\,O\,D-B\,l\,a\,n\,k\,G\,r\,o\,u\,p\,O\,D\right)}{\left(C\,o\,n\,t\,r\,o\,l\,G\,r\,o\,u\,p\,O\,D\right)-B\,l\,a\,n\,k\,G\,r\,o\,u\,p\,O\,D}\times 100\%$$

### Antibiotic Susceptibility and Hemolytic Activity

Antibiotic sensitivity and hemolytic activity are important indicators to evaluate the safety of probiotics. In this assay, the disc diffusion method was used to assess the antibiotic sensitivity of the isolated strains (Kapse et al., 2018). Specifically, the four isolated strains (1 × 10^8^ CFU/ml) were evenly spread onto LB agar plates with a sterile cotton swab. Then 12 drug-sensitive discs (ampicillin 10 μg, tetracycline 30 μg, gentamicin 10 μg, cefalexin 30 μg, enrofloxacin 10 μg, chloramphenicol 30 μg, norfloxacin 10 μg, erythromycin 15 μg, cefazolin 30 μg, vancomycin 30 μg, rifampin 5 μg, and lincomycin 2 μg) were used to evaluate the antibiotic sensitivity. After incubation at 37°C for 24 h, the inhibition zone diameter was measured using an electronic vernier caliper.

The hemolysis assay was processed according to the method (Liu et al., 2020). After overnight culture, all isolated strains were scribed on the LB agar plate containing sheep blood, and the *Staphylococcus aureus* BNCC186335 was used as a positive control. The hemolytic activity of the isolated strains was assessed via the presence of hemolytic rings after incubation at 37°C for 24 h.

### Antimicrobial Activities

Standard strain: *Escherichia coli* C83902, *S. aureus* BNCC186335, and *Salmonella enteritidis* NTNC13349 were provided by the state key laboratory of agricultural microbiology, Huazhong Agricultural University, Wuhan, China.

In the present study, the agar diffusion test was used to evaluate all isolated strains’ antimicrobial activity and three indicator pathogens (*E. coli* C83902, *S. aureus* BNCC186335, and *S. enteritidis* NTNC13349) were selected. LB liquid medium was used to raise these four strains, namely, Z1, Z2, J, and K, at 37°C for 24 h. Indicator pathogens (*E. coli*, *S. aureus*, and *S. enteritidis*) were activated in a 37°C LB liquid medium to rejuvenate. To test the antimicrobial activities, we refer to the method of Xia (Xia et al., 2019). Each of the three indicator bacteria (100 μl), *E. coli* (1.0 × 10^8^ CFU/ml), *S. aureus* (1.0 × 10^8^ CFU/ml), and *S. enteritidis* (1.0 × 10^8^ CFU/mL), were evenly spread in LB agar plates with a sterile cotton swab. The sterilized Oxford cups (round glass or metal tubes with an inner diameter of 6 mm, an outer diameter of 8 mm, and a height of 10 mm) were lightly pressed in LB agar plates. Respectively, the bacterial suspensions of all isolated strains (1.0 × 10^8^ CFU/mL) were injected into the Oxford cups with 100 μl. Finally, 100 μl of sterile water was added to the Oxford cup as a negative control group (Guo and Wang, 2017). There are three parallel spots for each strain. A vernier caliper measured inhibition areas after incubating at 37°C for 24 h.

### Autoaggregation Assay

The overnight cultures of isolated strains were centrifuged at 6,000 × *g* for 10 min at 4°C. Subsequently, the obtained bacterial cells were washed three times via PBS, and the concentration was adjusted to 10^8^ CFU/ml. After incubation of bacterial suspension at 37°C, the optical density (OD600) values were measured at 2, 4, 8, 16, and 28 h. The autoaggregation ability was described as follows:

$$A\,u\,t\,o-a\,g\,g\,r\,e\,g\,a\,t\,i\,o\,n\%=\left(1-\frac{A\,x}{A\,0}\right)\times 100\%$$

where A0 is the optical density at 0 h, and Ax is the optical density at ×h (2, 4, 8, 16, 28 h).

### Cell Surface Hydrophobicity Assay

The overnight cultures of isolated strains were centrifuged at 10,000 × *g* for 3 min at 4°C. Afterward, the obtained bacterial cells were washed three times using PBS, and then OD600 optical density values of the isolated strains were adjusted to 0.50 ± 0.05. An aliquot (3 ml) of bacterial suspension was combined with 3 ml of hexadecane and vortexed for 5 min at high speed. The mixture was allowed to stand for 1 h, and then the organic solvent was discarded to obtain an aqueous phase. The percentage of surface hydrophobicity was described as follows:

$$H\,y\,d\,r\,o\,p\,h\,o\,b\,i\,c\,i\,t\,y\%=\left(1-\frac{A\,x}{A\,0}\right)\times 100\%$$

where A0 represents the optical density at 0 h, and Ax represents the optical density at ×h.

### Statistical Analysis

All data were analyzed by one-way analysis of variance using SPSS 17.0 software. All data were expressed in mean ± SD, where SD is the standard deviation. A level of probability value (*p* < 0.05) was considered statistically significant.

## Results

### Species Isolation and Identification of the Bacteria

**FIGURE 1:**
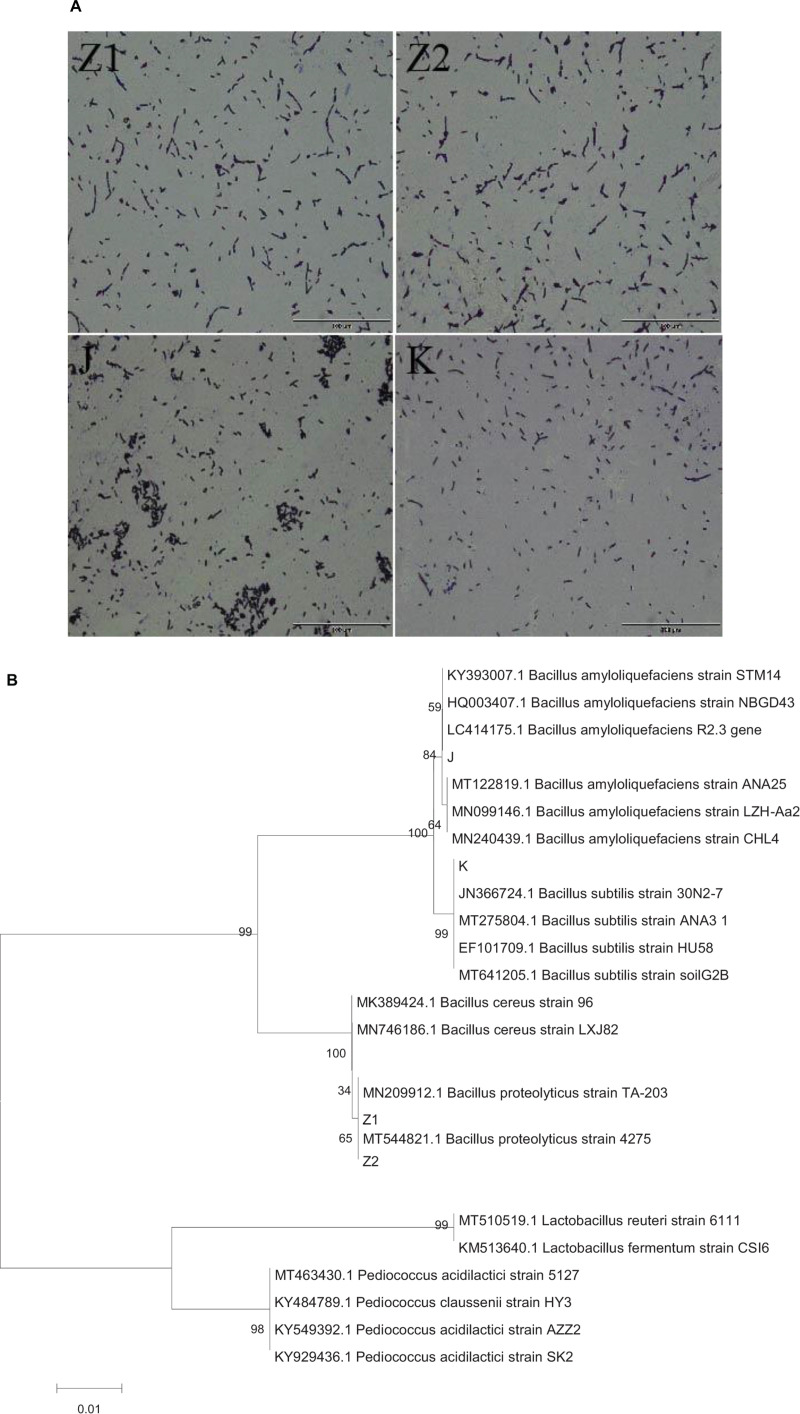
**(A)** The diagram shows the results of Gram staining for four isolates: Z1 and Z2 (*Bacillus proteolyticus*), J (*Bacillus amyloliquefaciens*), and K (*Bacillus subtilis*). **(B)** Phylogenetic tree constructed by using a neighbor-joining method on the basis for 16S rRNA gene sequences. J (*Bacillus amyloliquefaciens*), K (*B. subtilis*), Z1 and Z2 (*B. proteolyticus*).

After screening milky white, large, and developed colonies on LB agar, 40 *Bacillus* strains were identified (Supplementary Figure 1). Unfortunately, following many inoculations, some strains showed a considerable decrease in vitality. So, we only selected stable and well-produced strains (Z1, Z2, J, K) for further study to avoid repetition. All the isolated strains were Gram-positive rod-shaped (Figure 1A) and catalase negative (Table 2). The phylogenic tree was constructed based on the 16S rRNA gene, using the neighbor-joining method with MEGA 7 software (Figure 1B).

**TABLE 2:** Biochemical characterization of bacterial isolates.

**Biochemical**	**Z1**	**Z2**	**J**	**K**
Sodium citrate	+	+	+	+
Propionic acid	+	+	−	−
Fructose	+	+	+	+
Mannitol	−	−	+	+
Mannose	−	+	+	+
Galactose	−	−	−	−
Inulin	−	−	−	−
Rhamnose	−	−	−	−
Lactose	−	−	+	−
Myo-inositol	−	−	−	−
Salicin	−	−	−	−
Urea	−	−	−	−
Hydrogen sulfide	−	−	−	−
Dextrose	+	+	−	+
Sucrose	+	+	−	+
Xylose	−	+	−	−
Sorbitol	−	−	+	+
Catalase	−	−	−	−

*“+” positive reaction and “−” negative reaction.*

*The results were based on a bacterial biochemical assay kit (Qingtao Haibo Biotechnology Co., Ltd.).*

### Results of Antimicrobial Resistance Genes Tests

None of the isolated strains had tetracycline resistance genes, according to the findings. *tet(K), tet(L), tet(M), tet(O)* and vancomycin *van(A), van(B)*.

### Hydroxyl and DPPH Radical Scavenging Activity

**FIGURE 2:**
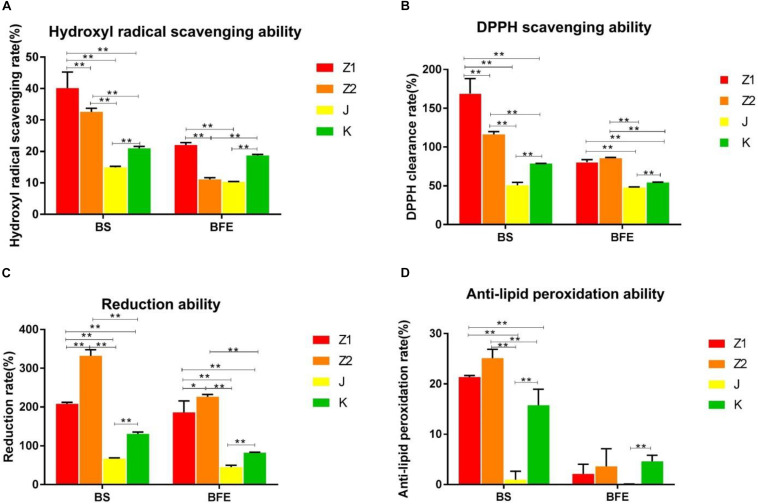
**(A,B)** The scavenging ability of BS and BFE on hydroxyl radicals and DPPH. **(C,D)** The results of lipid peroxidation reducing ability inhibitory activity and anti-lipid peroxidation ability of BS and BFE. BS, bacterial saline suspension; BFE bacteria-free extract. The data were expressed as the mean ± SD. *P < 0.05, **P < 0.01.

The radical scavenging activity of the isolated strains is shown in Figures 2A,B. The findings indicated that all of the isolated strains had strong radical scavenging activity and that various strains had high species specificity. The antioxidant capacity of the bacterial suspension was much higher than that of the nonbacterial extract. Specifically, the scavenging rate of the isolated strains for hydroxyl and DPPH radical were 14.67–46.05% and 46.15–190.04%, respectively. Interestingly, Z1 showed the highest hydroxyl and DPPH radicals scavenging activity among all the isolated strains (Figures 2A,B). On the contrary, J had the weakest scavenging ability for hydroxyl radical and DPPH radical (Figures 2A,B).

### Reducing Power and Lipid Peroxidation Inhibition Activity

The lipid peroxidation inhibitory activity and reduction ability of different strains were similar to free radical scavenging activity results. The inhibition rate of lipid peroxidation of the isolated strains varied from 2.9 to 26.77%. The results indicated that Z2 had the highest lipid peroxidation inhibition activity with an inhibition rate of 26.77%, while J had the lowest inhibition rate of 2.9% (Figure 2D). In addition, Z2 showed the most potent reducing power compared with other strains (Figure 2C).

### Autoaggregation and Cell Surface Hydrophobicity

**FIGURE 3:**
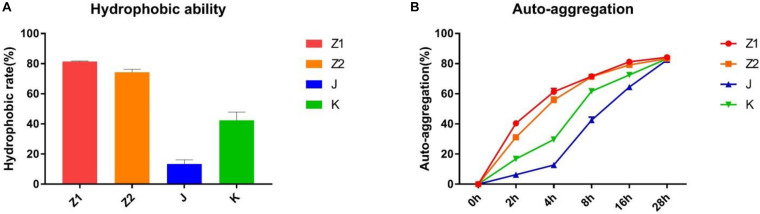
The results of autoaggregation and cell surface hydrophobicity experiment. **(A)** Hydrophobic ability. **(B)** Autoaggregation ability.

The ability of different strains to customize was very variable in the current investigation. The percentage hydrophobicity for the isolated strains was in the range of 14.40–81.92% (Figure 3A) for xylene. The agglutination ability for the isolated strains was in the span of 6.98–84.78% (Figure 3B). The maximum hydrophobic rate was observed among all the strains in Z1 (81.92%), while J exhibited the minimum hydrophobic rate (14.40%).

### Antibacterial Tests *in vitro*

**FIGURE 4:**
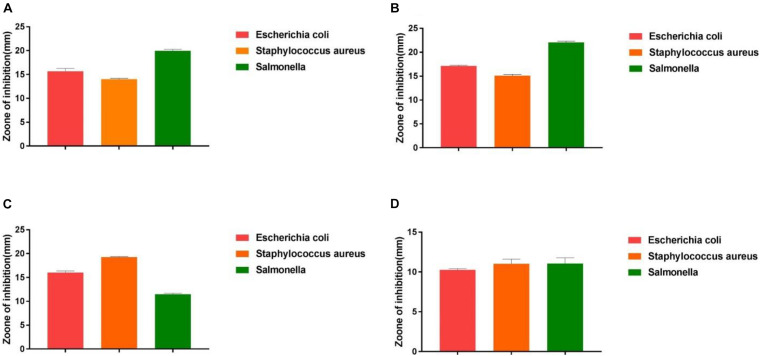
The results of the antibacterial experiment. **(A)** The result shows the antimicrobial capacity of Z1. **(B)** The result shows the antimicrobial capacity of Z2. **(C)** The result shows the antimicrobial capacity of J. **(D)** The result shows the antimicrobial capacity of K.

The antimicrobial activity of the isolated strains was evaluated according to the inhibition zone diameter. As shown in Figure 4, all the isolated strains exhibited inhibitory activities against *S. enteritidis*, *S. aureus*, and *E. coli*. However, different isolated strains exhibited different antimicrobial activity against different pathogens. Among all the isolated strains, Z2 exhibited the highest antimicrobial activity to *E. coli*, followed by Z1 and J, while J showed more significant inhibition to *S. aureus*, with a diameter of 19.20 mm. Z2 exhibited the strongest antimicrobial activity against *S. enteritidis*, with an inhibitory zone diameter of 22.13 mm.

### Antibiotic Susceptibility Assay and Hemolytic Activity

Our results showed that all the isolated strains suggested lower antibiotic resistance (Table 3). In addition, all the isolated strains were γ-hemolysis (no zone effect), and the *S. aureus* results in β hemolysis (blood lysis zones).

**TABLE 3:** The antibiotic susceptibility results of all strains.

**Antibiotics**	**Z1**	**Z2**	**J**	**K**
Ampicillin	R	R	S	R
Tetracycline	S	S	S	S
Gentamicin	S	S	S	S
Cefalexin	S	S	S	I
Enrofloxacin	S	S	S	S
Chloramphenicol	S	R	S	S
Norfloxacin	R	S	S	S
Erythromycin	I	I	S	I
Cefazolin	R	R	S	R
Vancomycin	S	S	S	S
Rifampin	R	R	R	R
Lincomycin	R	R	R	R

*S, sensitive; R, resistant; I, intermediate.*

*The test result was based on NCCLS (Wayne, 2003).*

### Heat Resistance, Acid, and Bile

**FIGURE 5:**
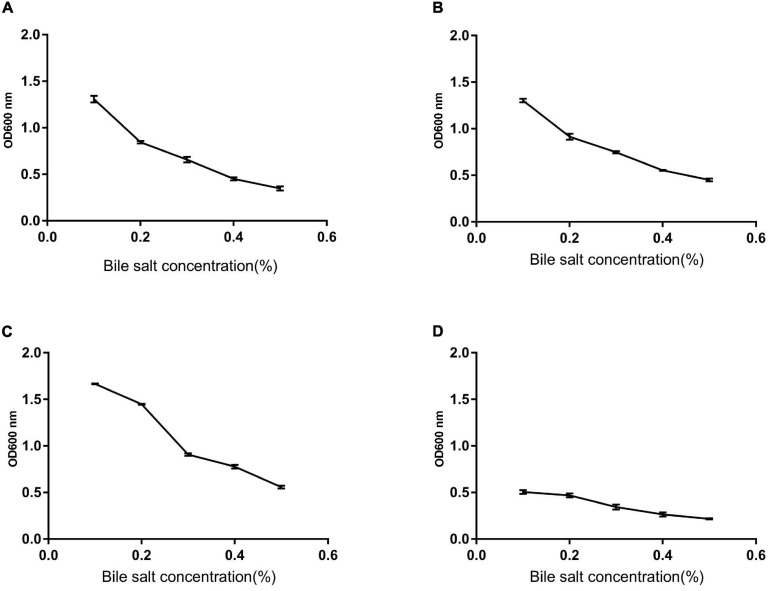
The survival rate of strains under different bile salt concentrations. **(A)** The result determines tolerance to bile salts of Z1. **(B)** The result determines tolerance to bile salts of Z2. **(C)** The result determines tolerance to bile salts of J. **(D)** The result determines tolerance to bile salts of K.

**FIGURE 6:**
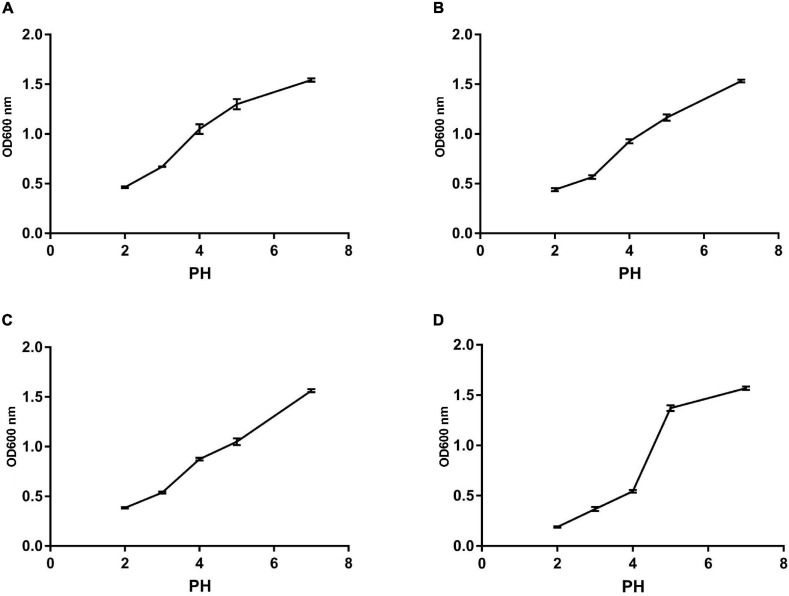
The survival rate of different strains under different acid conditions. **(A)** The result determines tolerance to acid conditions of Z1. **(B)** The result determines tolerance to acid conditions of Z2. **(C)** The result determines tolerance to acid conditions of J. **(D)** The result determines tolerance to acid conditions of K.

We monitored the survival rate of the isolates at various temperatures, acid, and bile salt at different concentrations in order to study their effect. As shown in Figures 5, 6, the survival rate of isolated strains gradually decreased with increased acid and bile concentrations. All the isolated strains had survival rates over 50 and 60% at pH 3.0 and 0.3% bile concentrations. Additionally, strain Z1 had the most substantial resistance to acid, with a survival rate of 53% at pH 3.0, while strain J had the highest tolerance to bile salts, with a survival rate of 66.7% at pH 3.0. On the other hand, the survival rate of the isolated strains remained above 80% at 40–80°C, while the survival rate decreased sharply at 80–100°C.

### The Resistance of Isolates to H_2_O_2_

**FIGURE 7:**
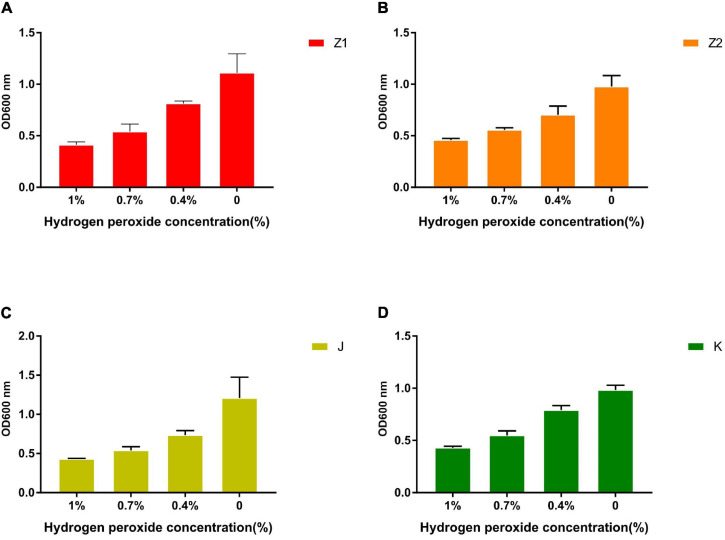
The tolerance ability of all strains to 1, 0.7, 0.4, and 0% concentration of H_2_O_2_ solution. **(A)** The result demonstrates the different concentrations of H_2_O_2_ tolerance of Z1. **(B)** The result demonstrates the different concentrations of H_2_O_2_ tolerance of Z2. **(C)** The result demonstrates the different concentrations of H_2_O_2_ tolerance of J. **(D)** The result demonstrates the different concentrations of H_2_O_2_ tolerance of K.

The growth of the isolates under different hydrogen peroxide concentrations is shown in Figure 7. The results showed that the tolerance of strains to H_2_O_2_ is concentration dependent. All the isolated strains showed strong resistance to 0.4 mM H_2_O_2_. However, the survival rates of the isolates were gradually dropped with the increase in H_2_O_2_ concentration. The survival rate of all the isolates was less than 10% when subjected to 1.0 mM H_2_O_2_ for 8 h.

## Discussion

It is well known that animals are susceptible to oxidative stress due to environmental, nutritional, and physiological factors in the breeding process (Kara et al., 2005). Moreover, heat stress in broiler chickens and animals might decrease production performance (Tang et al., 2016). Therefore, many measures have been performed to improve the antioxidant capacity of animals to mitigate the oxidative adverse effects of stress. Conventional synthetic antioxidants have been gradually restricted to use due to various toxic effects (Pattono et al., 2009). On the other hand, probiotics have gotten much attention because of their many probiotic characteristics (Rehaiem et al., 2014). Previous research has shown that the yak intensely adaptable to intense ultraviolet rays, cold, and oxygen deficiency (Li et al., 2018). In the current study, we attempted to isolate *Bacillus* from the feces of yaks and explore its antioxidant properties *in vitro* by Gram staining, biochemical assays (Bergey’s manual of determinative bacteriology) and 16S rRNA analysis. Our results firstly revealed that Z1 and Z2 (*B. proteolyticus*), J (*B. amyloliquefaciens*), and K (*B. subtilis*) isolated from yaks have good free radical scavenging ability and probiotic properties.

Furthermore, probiotics can also stimulate the host’s antioxidant system to improve antioxidant enzyme levels (Sanders et al., 2019). More notably, probiotics can also produce multiple metabolites with antioxidant activity. However, not all probiotics have antioxidant properties due to the high heterogeneity of strains (Wang et al., 2017). In this study, we attempted to screen out *Bacillus* with antioxidant properties *in vitro*. Our results suggested that the isolated strains have high DPPH radical and hydroxyl radical scavenging ability with higher hydrogen peroxide tolerance, lipid peroxidation inhibition activity, and reducing power. Specifically, Z1 (*B. proteolyticus*) showed the highest DPPH and hydroxyl radical scavenging activity, while Z2 (*B. proteolyticus*) was better in reducing power and lipid peroxidation inhibition.

Hydrogen peroxide can penetrate most of the cell membrane and react with iron in the cell to form hydroxyl radicals (Brackman and Havinga, 1955). Therefore, hydrogen peroxide is more cytotoxic than superoxide anion radicals that cannot penetrate cell membranes. The catalase’s main biological function is to accelerate the decomposition of hydrogen peroxide in cells and prevent the further production of highly toxic hydroxyl radicals (Li et al., 2011). The results are also consistent with previous findings where Z1, Z2, J, and K exhibited strong tolerance to hydrogen peroxide. Hence, the strains isolated from yaks contained strong antioxidant capacity, consistent with the extremely harsh living environment of yaks.

Stomach and intestine can secrete a large amount of gastric juice and bile into the body every day, tolerance of the acidic environment in the stomach and the bile salt environment in the intestine is the foundation on which probiotics exert their positive effects on the host. At present, deactivation of bacteria during processing and transport is now one of the most serious problems for probiotics (Liu et al., 2020). However, *Bacillus* strain is stronger against stress, which multiplies quickly and tenacious vitality. More importantly, *Bacillus* strains can form endophytic resistant spores to resist high temperature, acid, and alkali polar environments (Kapse et al., 2018). In the current study, all the isolated strains showed great thermal ability, as well as varying degrees of tolerance to acid and bile. Concretely, *B. amyloliquefaciens* (J) exhibited the strongest bile salt tolerance in 0.3% concentration compared with *B. subtilis* (K). While Z1 (*B. proteolyticus*) showed the highest acidic ability at pH less than 4.0 than *B. subtilis* (K). These results are consistent with the Kapse findings.

Antibiotics are widely used to improve growth performance and treat bacterial diseases (Li et al., 2019b). However, the misuse of antibiotics may increase the emergence of antibiotic-resistant bacteria and dysbacteriosis. Previous studies have shown that antibiotic resistance can be overwhelming among microbial communities, and even some probiotics may have resistance genes. In addition, antibiotic resistance can also spread to humans by the food supply chain and lead to a significant threat to human health and food safety (Verraes, 2013). According to FAO/WHO regulations, probiotics must be supported by safety evidence before they are used in clinical practice (FAO, 2002). We also tested the antagonistic effect of the isolated strain on *E. coli* C83902, *S. aureus* BNCC186335, and *S. enteritidis* NYNC13349 to evaluate the antibacterial effect. Consistent with previous research (Redman et al., 2014), antimicrobial resistance genes, antibiotic susceptibility, and hemolytic activity were used to demonstrate the safety of the four strains identified in the experiment. The antimicrobial resistance genes and hemolytic activity tests revealed that none of the four strains was positive. Furthermore, all strains were active against Gram-positive and negative pathogens, although their effectiveness varied. If these three or more probiotics are combined, it may provide better clinical effects. However, further research is required before the clinical applications of these strains.

The autoaggregation and cell surface hydrophobicity of probiotics are both important features. Adhesion between cells causes autoaggregation, which is closely linked to intestinal adhesion and the production of beneficial biofilms (Agostiano et al., 1993). Generally, the ability of adhesion between probiotics and intestines and the formation of beneficial biofilms are positively correlated with the ability of agglutination. In addition, probiotic biofilms can also reduce the colonization of pathogenic bacteria in the intestines and improve the resistance of the gut to biological, chemical, and physical attacks. Prior studies have suggested that the adhesion of the microorganisms is closely related to the surface properties of the bacteria (O’Mahony et al., 2005). The cell surface hydrophobicity is the main factor that determines the nonspecific adhesion of bacteria to the host surface. Bacterial cells can interact with digestive tract epithelial cells via nonspecific noncovalent weak interactions called hydrophobic interactions, and the force among them is positively correlated to hydrophobicity (Kamberi et al., 2004; Israelachvili, 2005; Petrie et al., 2011). Therefore, the method of microbial adhesion to hydrocarbons was used to evaluate the cell surface hydrophobicity. Our results demonstrated that the isolated strains were highly capable of cell surface hydrophobicity and autoaggregation (the highest of which were Z1 and Z2), suggesting that the isolated strains may have high adhesion to intestinal mucosal cells.

## Conclusion

The present study revealed that the isolates from yak had strong antioxidant and probiotic activity. Moreover, these strains proved safe through *in vitro* experiments with restraining ability against the proliferation of pathogenic bacteria. These abilities imply an important role in bacterial diseases. This was the first study to reveal the probiotic potential of *B. proteolyticus* and *B. amyloliquefaciens* isolated from yaks.

## Data Availability Statement

The original contributions presented in the study are included in the article/Supplementary Material, further inquiries can be directed to the corresponding author/s.

## Author Contributions

ZZ, XH, and JL conceived and designed the experiments. FL, YZ, ZH, YW, KL, and YB contributed sample collection and reagents preparation. MF and MI revised the manuscript. ZZ and XH wrote the manuscript. All authors contributed to the article and approved the submitted version.

## Conflict of Interest

YB was employed by company Tibet Autonomous Region Biological Drug Manufacturing Plant. The remaining authors declare that the research was conducted in the absence of any commercial or financial relationships that could be construed as a potential conflict of interest.

## Publisher’s Note

All claims expressed in this article are solely those of the authors and do not necessarily represent those of their affiliated organizations, or those of the publisher, the editors and the reviewers. Any product that may be evaluated in this article, or claim that may be made by its manufacturer, is not guaranteed or endorsed by the publisher.

## Supplementary Material

Supplementary Figure 1 This diagram shows the results of the morphology of the four isolated strains. Z1 and Z2 (*Bacillus proteolyticus*); J (*Bacillus amyloliquefaciens*); K (*Bacillus subtilis*).

## References

[B1] Aarestrup F. M. Agerso Y. Gerner-Smidt P. Madsen M. Jensen L. B. (2000). Comparison of antimicrobial resistance phenotypes and resistance genes in Enterococcus faecalis and Enterococcus faecium from humans in the community, broilers, and pigs in Denmark. *Diagn. Microbiol. Infect. Dis.* 37 127–137. 10.1016/S0732-8893(00)00130-9 10863107

[B2] Agostiano A. Monica M. D. Palazzo G. Trotta M. (1993). Chlorophyll a auto-aggregation in water rich region. *Biophys. Chem.* 47 193–202. 10.1016/0301-4622(93)85036-H

[B3] Areskogh D. Henriksson G. (2011). Fenton’s reaction: a simple and versatile method to structurally modify commercial lignosulphonates. *Nord. Pulp Paper Res. J.* 26 90–98. 10.3183/npprj-2011-26-01-p090-098

[B4] Asad N. R. Asad L. M. B. O. Silva A. B. Felzenszwalb I. Leitão A. C. (1998). Hydrogen peroxide induces protection against lethal effects of cumene hydroperoxide in *Escherichia coli* cells: an Ahp dependent and OxyR independent system? *Mutat. Res.* 407 253–259. 10.1016/S0921-8777(98)00010-X 9653451

[B5] Brackman W. Havinga E. (1955). The oxidation of phenols with copper−amine catalysts and its relation to the mode of action of tyrosinase IV. Relations between hydrogen peroxide and the catalytic oxidation of phenols. *Recl. Trav. Chim. Pays Bas* 74 1100–1106. 10.1002/recl.19550740908

[B6] Dutka-Malen S. Evers S. Courvalin P. (1995). Detection of glycopeptide resistance genotypes and identification to the species level of clinically relevant enterococci by PCR. *J. Clin. Microbiol.* 33 24–27. 10.1128/jcm.33.1.24-27.1995 7699051 PMC227872

[B7] Eiamphungporn W. Nakjarung K. Prapagdee B. Vattanaviboon P. Mongkolsuk S. (2003). Oxidant-inducible resistance to hydrogen peroxide killing in *Agrobacterium tumefaciens* requires the global peroxide sensor-regulator OxyR and KatA. *FEMS Microbiol. Lett.* 225 167–172. 10.1016/S0378-1097(03)00511-1 12900037

[B8] FAO (2002). *WHO Working Group Report on Drafting Guidelines for the Evaluation of Probiotics in Food*. London: FAO, 30.

[B9] Guo L. Wang C. (2017). Optimized production and isolation of antibacterial agent from marine *Aspergillus flavipes* against Vibrio harveyi. *3 Biotech* 7:383. 10.1007/s13205-017-1015-z 29134160 PMC5661027

[B10] Hossain K. F. B. Hosokawa T. Saito T. Kurasaki M. (2020). Amelioration of butylated hydroxytoluene against inorganic mercury induced cytotoxicity and mitochondrial apoptosis in PC12 cells via antioxidant effects. *Food Chem. Toxicol.* 146:111819. 10.1016/j.fct.2020.111819 33091556

[B11] Israelachvili J. (2005). Differences between non-specific and bio-specific, and between equilibrium and non-equilibrium, interactions in biological systems. *Q. Rev. Biophys.* 38 331–337. 10.1017/S0033583506004203 16780605

[B12] Kamberi M. Chung P. DeVas R. Li L. Li Z. Ma X. S. (2004). Analysis of non-covalent aggregation of synthetic hPTH (1–34) by size-exclusion chromatography and the importance of suppression of non-specific interactions for a precise quantitation. *J. Chromatogr. B Analyt. Technol. Biomed. Life Sci.* 810 151–155. 10.1016/j.jchromb.2004.07.026 15358319

[B13] Kapse N. G. Engineer A. S. Gowdaman V. Wagh S. Dhakephalkar P. K. (2018). Genomics functional annotation of the genome unravels probiotic potential of *Bacillus coagulans* HS243. *Genomics* 111 921–929. 10.1016/j.ygeno.2018.05.022 29859262

[B14] Kara H. Karataş F. Canatan H. (2005). Effect of single dose cadmium chloride administration on oxidative stress in male and female rats. *Turk. J. Vet. Anim. Sci.* 29 37–42.

[B15] Klimek J. Schaap A. P. Kimura T. (1982). Inhibition of lipid peroxidation by paraquat: site of inhibition in the cytochrome P-450-dependent steroid hydroxylase system from bovine adrenal cortex mitochondria. *Biochem. Biophys. Res. Commun.* 107 499–505. 10.1016/0006-291X(82)91519-4 6215039

[B16] Li A. Jiang X. Wang Y. Zhang L. Zhang H. Mehmood K. (2019a). The impact of *Bacillus subtilis* 18 isolated from Tibetan yaks on growth performance and gut microbial community in mice. *Microb. Pathog.* 128 153–161. 10.1016/j.micpath.2018.12.031 30583023

[B17] Li A. Wang Y. Pei L. Mehmood K. Li K. Qamar H. (2019b). Influence of dietary supplementation with *Bacillus velezensis* on intestinal microbial diversity of mice. *Microb. Pathog.* 136:103671. 10.1016/j.micpath.2019.103671 31437575

[B18] Li J. Guo D. Zhou D. Wu X. (2011). Teaching veterinary internal medicine in China. *J. Vet. Med. Educ.* 38 194–198. 10.3138/jvme.38.2.194 22023928

[B19] Li J. Li K. Shahzad M. Han Z. Nabi F. Gao J. (2015). Seroprevalence of Bluetongue virus in domestic yaks (*Bos grunniens*) in Tibetan regions of China based on circulating antibodies. *Trop. Anim. Health Prod.* 47 1221–1223. 10.1007/s11250-015-0853-0 26017752

[B20] Li K. Li Z. Zeng Z. Li A. Mehmood K. Shahzad M. (2020). Prevalence and molecular characterization of *Cryptosporidium* spp. in yaks (*Bos grunniens*) in Naqu, China. *Microb. Pathog.* 144:104190. 10.1016/j.micpath.2020.104190 32272216

[B21] Li K. Shahzad M. Zhang H. Jiang X. Mehmood K. Zhao X. (2018). Socio-economic burden of parasitic infections in yaks from 1984 to 2017 on Qinghai Tibetan Plateau of China—a review. *Acta Trop.* 183 103–109. 10.1016/j.actatropica.2018.04.011 29626434

[B22] Liu J. Wang Y. Li A. Iqbal M. Zhang L. Pan H. (2020). Probiotic potential and safety assessment of *Lactobacillus* isolated from yaks. *Microb. Pathog.* 145:104213. 10.1016/j.micpath.2020.104213 32333954

[B23] Moné Y. Ribou A. C. Cosseau C. Duval D. Théron A. Mitta G. (2011). An example of molecular co-evolution: reactive oxygen species (ROS) and ROS scavenger levels in *Schistosoma mansoni*/*Biomphalaria glabrata* interactions. *Int. J. Parasitol.* 41 721–730. 10.1016/j.ijpara.2011.01.007 21329695

[B24] Mukherjee N. Mukherjee S. Saini P. Roy P. Sinha Babu S. P. (2014). Antifilarial effects of polyphenol rich ethanolic extract from the leaves of *Azadirachta indica* through molecular and biochemical approaches describing reactive oxygen species (ROS) mediated apoptosis of *Setaria cervi*. *Exp. Parasitol.* 136 41–58. 10.1016/j.exppara.2013.11.006 24275557

[B25] O’Mahony R. Basset C. Holton J. Vaira D. Roitt I. (2005). Comparison of image analysis software packages in the assessment of adhesion of microorganisms to mucosal epithelium using confocal laser scanning microscopy. *J. Microbiol. Methods* 61 105–126. 10.1016/j.mimet.2004.11.020 15676201

[B26] Oyaizu M. (1988). Antioxidative activities of browning products of glucosamine fractionated by organic solvent and thin-layer chromatography. *Nippon Shokuhin Kogyo Gakkaishi* 35 771–775. 10.3136/nskkk1962.35.11_771

[B27] Pattono D. Battaglini L. M. Barberio A. De Castelli L. Valiani A. Varisco G. (2009). Presence of synthetic antioxidants in organic and conventional milk. *Food Chem.* 115 285–289. 10.1016/j.foodchem.2008.11.055

[B28] Petrie K. Docoslis A. Vasic S. Kontopoulou M. Morgan S. Ye Z. (2011). Non-covalent/non-specific functionalization of multi-walled carbon nanotubes with a hyperbranched polyethylene and characterization of their dispersion in a polyolefin matrix. *Carbon N. Y.* 49 3378–3382. 10.1016/j.carbon.2011.04.001

[B29] Pires B. R. B. Panis C. Alves V. D. Herrera A. C. S. A. Binato R. Pizzatti L. (2019). Label-free proteomics revealed oxidative stress and inflammation as factors that enhance chemoresistance in luminal breast cancer. *Oxid. Med. Cell. Longev.* 2019:5357649. 10.1155/2019/5357649 31485295 PMC6702830

[B30] Redman M. G. Ward E. J. Phillips R. S. (2014). The efficacy and safety of probiotics in people with cancer: a systematic review. *Ann. Oncol.* 25 1919–1929. 10.1093/annonc/mdu106 24618152

[B31] Rehaiem A. Belgacem Z. B. Edalatian M. R. Martínez B. Rodríguez A. Manai M. (2014). Assessment of potential probiotic properties and multiple bacteriocin encoding-genes of the technological performing strain *Enterococcus faecium* MMRA. *Food Control* 37 343–350. 10.1016/j.foodcont.2013.09.044

[B32] Ramos-Trujillo E. Pérez-Roth E. Méndez-Alvarez S. Claverie-Martín F. (2003). Multiplex PCR for simultaneous detection of enterococcal genes vanA and vanB and staphylococcal genes mecA, ileS-2 and femB. *Int. Microbiol.* 6 113–115. 10.1007/s10123-003-0118-z 12802618

[B33] Sanders M. E. Merenstein D. J. Reid G. Gibson G. R. Rastall R. A. (2019). Author correction: probiotics and prebiotics in intestinal health and disease: from biology to the clinic (nature reviews gastroenterology & hepatology, (2019), 16, 10, (605-616), 10.1038/s41575-019-0173-3). *Nat. Rev. Gastroenterol. Hepatol.* 16:642. 10.1038/s41575-019-0199-6 31399728

[B34] Tang S. Yin B. Song E. Chen H. Cheng Y. Zhang X. (2016). Aspirin upregulates αb-Crystallin to protect the myocardium against heat stress in broiler chickens. *Sci. Rep.* 6:37273. 10.1038/srep37273 27857180 PMC5114548

[B35] Verraes C. Van Boxstael S. Van Meervenne E. Van Coillie E. Butaye P. Catry B. (2013). Antimicrobial resistance in the food chain: a review. *Int. J. Environ. Res. Public Health* 10 2643–2669. 10.3390/ijerph10072643 23812024 PMC3734448

[B36] Wang Y. Li A. Jiang X. Zhang H. Mehmood K. Zhang L. (2018). Probiotic potential of *Leuconostoc pseudomesenteroides* and *Lactobacillus Strains* isolated from yaks. *Front. Microbiol.* 9:2987. 10.3389/fmicb.2018.02987 30564222 PMC6289064

[B37] Wang Y. Wu Y. Wang B. Xu H. Mei X. Xu X. (2019). *Bacillus amyloliquefaciens* SC06 protects mice AGAINST high-fat diet-induced obesity and liver injury via regulating host metabolism and gut microbiota. *Front. Microbiol.* 10:1161. 10.3389/fmicb.2019.01161 31191487 PMC6547872

[B38] Wang Y. Wu Y. Wang Y. Xu H. Mei X. Yu D. (2017). Antioxidant properties of probiotic bacteria. *Nutrients* 9:521. 10.3390/nu9050521 28534820 PMC5452251

[B39] Wayne P. (2003). *National Committee for Clinical Laboratory Standards: Performance Standards for Antimicrobial Susceptibility Testing. NCCLS Doc. M100-S13 (M2 A8), USA*.

[B40] Wu H. Fang J. Tang L. Lu P. Xu H. Zhao Y. (2014). Quality evaluation of astragali radix based on DPPH radical scavenging activity and chemical analysis. *Chin. Herb. Med.* 6 282–289. 10.1016/s1674-6384(14)60043-5

[B41] Xia Y. Qin S. Shen Y. (2019). Probiotic potential of Weissella strains isolated from horse feces. *Microb. Pathog.* 132 117–123. 10.1016/j.micpath.2019.04.032 31009656

