# Supplementary figures and images for: Probiotic Properties of Bacillus proteolyticus Isolated From Tibetan Yaks, China
Source: Front Microbiol. 2021 Aug 17;12:649207. doi: 10.3389/fmicb.2021.649207 (PMC8416245; doi:10.3389/fmicb.2021.649207)

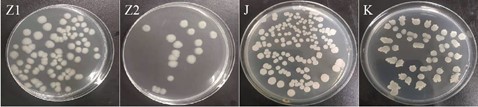

Supplement: Supplementary Figure 1 — This diagram shows the results of the morphology of the four isolated strains. Z1 and Z2 (Bacillus proteolyticus); J (Bacillus amyloliquefaciens); K (Bacillus subtilis). [file Image_1.jpg]
